# Noise in the Intensive Care Unit: A Narrative Review of Its Characteristics, Clinical Impact, and Reduction Strategies

**DOI:** 10.1155/ccrp/4782724

**Published:** 2026-04-13

**Authors:** Charikleia S. Vrettou, Panagiotis T. Koliotsis, Spyretta Golemati, Giorgos Mastorakis, Vassiliki Karaviti, Sofia Mavromati, Maria Tagara, Maria P. Papadopoulou, Ioanna Dimopoulou

**Affiliations:** ^1^ First Department of Critical Care Medicine, Evangelismos Hospital, Medical School, National & Kapodistrian University of Athens, Athens, 10676, Greece, uoa.gr; ^2^ Laboratory of Physical Geography and Environmental Impacts, School of Rural, Surveying and Geoinformatics Engineering, National Technical University of Athens, Zografou Campus, Athens 15780, Greece, ntua.gr

**Keywords:** environmental exposure, intensive care units, noise, patient safety, workplace safety

## Abstract

**Aims:**

Noise in the intensive care unit (ICU) is an environmental stressor affecting both patients and healthcare professionals. This narrative review synthesizes evidence from over 2 decades of research regarding ICU noise levels, sources, acoustic characteristics, and clinical impact.

**Design:**

Narrative review.

**Data Sources:**

A literature search was conducted in PubMed for studies published between January 2000 and July 2025. Search terms included combinations of ICU, intensive care, noise, sound levels, alarm fatigue, acoustic environment, delirium, sleep disruption, burnout, and hospital design. Reference lists of relevant reviews and original studies were screened.

**Review Methods:**

Eligible publications included original research, simulation studies, systematic reviews, clinical guidelines, and qualitative reports focusing on adult ICUs. Only English‐language studies were included. Data were narratively synthesized to describe noise levels, sources, impacts, and mitigation strategies.

**Results:**

Sound levels in most ICUs routinely exceed 55–60 dBA, with peak levels often surpassing 85–90 dBA—far above World Health Organization recommendations. Common noise sources include medical equipment alarms, staff activity, and environmental design features such as open layouts and reflective surfaces. Acoustic characteristics such as unpredictability and poor nighttime attenuation amplify stress. In patients, noise contributes to sleep fragmentation, circadian disruption, increased sedation needs, delirium, and adverse psychological outcomes. Among staff, excessive noise impairs communication, increases cognitive load, and contributes to fatigue and burnout. While various architectural, behavioral, and bundled interventions have shown promise, most demonstrate limited long‐term effectiveness.

**Conclusion:**

Despite clear guidelines, ICU noise remains inadequately managed due to systemic barriers, including cultural norms, infrastructural constraints, and a lack of enforcement.

**Impact**
• Noise control should be recognized as an essential patient safety and staff well‐being strategy.
• Sustainable mitigation requires integrated approaches combining design‐informed environments, behavioral engagement, and institutional accountability.

## 1. Introduction

Noise in the intensive care unit (ICU) is increasingly recognized as a persistent environmental hazard with wide‐ranging implications for patient care and staff well‐being [1, 2]. Defined as unwanted or disturbing sound, noise in the ICU arises from a combination of medical equipment, alarms, human activity, and architectural design flaws. Two major regulatory bodies have issued widely referenced recommendations for acceptable indoor noise levels in healthcare settings. The World Health Organization (WHO) advises that average daytime sound levels in general patient care areas should not exceed 35 dBA and that maximum nighttime levels should remain below 40 dBA, according to its Guidelines for Community Noise published in 1999 [2–4]. The U.S. Environmental Protection Agency (EPA), through its Office of Noise Abatement and Control, recommends slightly more lenient thresholds: average hospital noise levels of no more than 45 dBA during the day and 35 dBA at night [5]. Despite longstanding awareness, environmental noise remains a persistent and underaddressed stressor in these settings. Sound pressure levels (SPLs) in ICUs often surpass thresholds recommended by the WHO, contributing to a clinical environment that may negatively impact both patients and staff [6–10].

Beyond exceeding recommended thresholds, the clinical relevance of ICU noise lies in its acoustic characteristics rather than sound intensity alone. ICU soundscapes are dominated by intermittent, unpredictable, and high‐frequency noise peaks, such as alarms and staff activity, that are poorly attenuated during nighttime hours. In critically ill patients, sustained exposure to these stressors disrupts sleep continuity and circadian regulation, activates neuroendocrine stress responses, and may contribute to delirium and increased sedation needs. For healthcare professionals, excessive background noise impairs speech intelligibility, cognitive performance, and communication, contributing to fatigue and burnout. Despite international guidelines and decades of research, ICU noise levels have remained largely unchanged. Most studies focus on documenting sound levels or testing isolated interventions with short follow‐up periods and heterogeneous measurement methods. Consequently, evidence remains fragmented, and there is limited consensus regarding which noise‐reduction strategies are most effective, sustainable, and feasible across ICU settings.

This narrative review aims to synthesize current evidence on ICU noise characteristics, sources, clinical impact on patients and staff, and available mitigation strategies, while highlighting persistent knowledge and implementation gaps that hinder effective noise control in clinical practice. In particular, this review highlights gaps related to the lack of standardized noise metrics, limited long‐term evaluation of interventions, insufficient prioritization of mitigation strategies, and persistent barriers to implementation in everyday ICU practice.

## 2. Methods

A literature search was conducted in PubMed to identify studies addressing noise in the ICU and its impact on patients, staff, and the clinical environment. Searches were limited to articles published in English between January 2000 and July 2025 and used combinations of the following terms: “intensive care,” “ICU,” “noise,” “sound levels,” “alarms,” “acoustic environment,” “sleep disruption,” “delirium,” “burnout,” and “hospital design”. Reference lists of relevant reviews and original studies were manually screened to identify additional publications.

Studies were included if they reported on adult ICU settings and addressed noise measurement, noise sources, clinical or occupational impact, or noise‐reduction interventions. We included original research articles, systematic and narrative reviews, clinical guidelines, simulation studies, and qualitative reports. Studies focusing exclusively on pediatric or neonatal populations, nonclinical acoustic environments, or non‐English publications were excluded.

## 3. Results

### 3.1. Methods for Identifying and Measuring Sound Level in the ICU

Sound levels in the ICU are typically quantified using the SPL, expressed in decibels (dB). SPL is a logarithmic measure that reflects the pressure of a sound relative to a standardized reference of 20 μPa, the threshold of human hearing in air. This metric allows for consistent and objective comparison of acoustic exposures in clinical environments [11].

A growing body of research has investigated the acoustic characteristics of ICUs, aiming to assess both the intensity and sources of noise through various methodologies. The methods employed for sound assessment are classified into four main categories: (1) objective measurements using calibrated sound level meters, (2) observational techniques for identifying noise sources, (3) the use of portable devices and smartphone‐based applications, and (4) indirect approaches assessing the physiological and psychological impact of noise exposure. The LAeq Index (A‐weighted equivalent continuous sound level) is now widely recognized as a scientifically validated metric for evaluating exposure to fluctuating or continuous acoustic environments. It is defined as the constant SPL that, over a specified time interval, would contain the same total acoustic energy as the actual varying SPL observed during that period. LAeq is measured in decibels [dB(A)], where the A‐weighting filter adjusts the measurement to reflect the human ear’s sensitivity by attenuating low and very high frequencies that are less perceptible to human hearing [11].

*L*_Aeq_ incorporates an energy‐based approach to acoustic analysis, as it relies on the integration of sound energy over time. The mathematical expression used to calculate *L*_Aeq_ is as follows:

(1) LAeq=10∗log101Τ ∫0Τ10LAt/10dt,

where ${L_{A}}^{\left(t\right)}$ is the instantaneous A‐weighted SPL at time t, measured in dB(A), and *T* is the total duration of the measurement period. In practice, *L*_Aeq_ is computed using specialized sound measurement instruments (sound level meters) that continuously integrate sound pressure values over time. The calculation is based on the logarithmic average of sound energy, making *L*_Aeq_ a more stable and reliable indicator compared to momentary values or singular metrics such as the maximum sound level (*L*_max_). The application of *L*_Aeq_ is of fundamental importance in numerous scientific and regulatory domains, including the design and acoustic assessment of indoor environments such as schools, hospitals, and office buildings, where maintaining acceptable noise levels is crucial for comfort and cognitive performance [12]. One of *L*_Aeq_’s key advantages lies in its adaptability across different time scales, allowing measurements to be taken over intervals ranging from a few seconds to 24 h or even annual averages. This flexibility enhances its usefulness in both short‐term noise monitoring and long‐term exposure assessments, which are strongly associated with health outcomes such as cardiovascular disease and sleep disturbances [4].

### 3.2. Sources and Characteristics of ICU Noise

ICU noise arises from a combination of technical, environmental, behavioral, and patient‐related factors. Primary sources include medical equipment and alarms, staff conversations and other human activities, and external environmental inputs, while architectural design features such as surface materials and unit layout significantly shape the acoustic environment [13–17]. In a 3‐month observational study, Park et al. [18] found that patients’ initial severity of illness, as measured by APACHE II scores, was significantly associated with higher ambient noise levels in ICU rooms.

**FIGURE 1:**
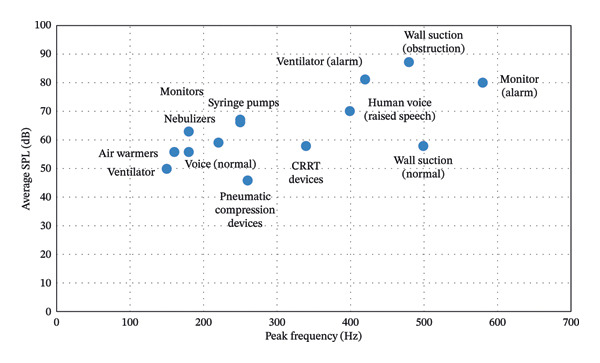
ICU sound sources, peak frequency, and sound level. SPL, sound pressure level; CRRT, continuous renal replacement therapy.

ICU bedsides are densely equipped with devices such as ventilators, monitors, and infusion pumps, many of which generate alarms. These devices contribute to a persistent low‐frequency acoustic baseline, punctuated by high‐intensity peaks [1, 15, 19–23]. Alarm sounds alone frequently exceed WHO‐recommended thresholds. Staff interactions, especially during handovers and bedside procedures, are also frequent sources of peak noise levels [24, 25]. Routine care activities, including repositioning patients, administering medication, and cleaning, add to the ambient sound burden. Elevated average noise levels are more common in units with higher patient turnover and lower nurse‐to‐patient ratios [26]. A summary of representative studies reporting ICU noise levels, dominant sound sources, and selected outcomes is presented in Table 1. These studies span diverse settings and methodologies yet consistently show sound levels exceeding international guidelines. Figure 1 illustrates average SPLs and peak frequencies of common ICU noise sources, highlighting the prominent acoustic burden of alarms, raised voices, and suction events.

**TABLE 1:** Typical noise levels in the intensive care across studies, notable noise sources, and major study results.

Study (country) (study design)	ICU type	Mean noise level (dBA)	Peak noise levels (dBA)	Notable sources	Major results
Tahvili et al. (United Kingdom) [98] (OS)	General ICU	44	87	Patient care, suctioning	Noise levels exceeded national and international guidelines
Song and Lee (China) [53] (OM)	General ICU	NR	73.9	Background equipment noise, voice	Mean levels exceeded WHO recommendations. Background noise was significantly correlated with vocal strain.
Imbriaco et al. (Italy) [64] (SiS)	Simulated ICU	45.3–53.5	98	Medical device alarms, nursing tasks, conversations	Identified staff‐generated noise as most disturbing and loudest events
Lucchini et al. (Italy) [99] (OS)	General ICU	54.6	85	Human activities	Noise levels exceeded WHO/EPA recommendations at nearly all time points
Leone et al. (United States) [100] (OS)	Medical/surgical ICU	52.5	NS	Clinical activity, monitors, alarms	Excess sound and light levels across multiple
Jung et al. (South Korea) [1] (OS)	Medical and surgical ICUs	54.4	91	Mechanical equipment, patient‐monitoring devices	ICU noise exceeded expected limits; structural interventions are needed to mitigate high noise levels
Terzi et al. (Turkey) [56] (OS)	Mixed ICUs	64.5	99	Alarms, staff activity	Noise levels in ICUs well above the recommended levels
Bani Younis et al. (Jordan) [101] (OS)	General ICU	63.9	102	Monitor alarms, staff talking	Negative correlation between nocturnal sound levels and perceived sleep quality
Aydın Sayılan et al. (Turkey) [33] (OS)	General ICU	66.5	78	Monitor alarms, staff conversation, mechanical sounds	All measured noise levels exceeded WHO limits and this adversely affects patients’ anxiety levels and sleep quality
Simons et al. (Netherlands) [34] (OM)	Mixed ICUs	54.0	86.0	Talking, alarms	Background noise had a negative impact on sleep quality
Garrido Galindo et al. (Colombia) [102] (OS)	General ICU	63.4	79.19	Clinical activities and alarms	Time of day exert is the greatest influence upon noise level
Hu et al. (China) [48] (OM)	General ICU	59.0	83.3	Alarms, staff conversations, equipment noise, patient care activities	Noise levels consistently exceeded WHO recommended limits
Darbyshire et al. (UK) [6] (OM)	General adult	52–59	127.9	Equipment and staff activity	All ICUs had sound levels greater than WHO recommendations, side room needed full equipment shutdown to meet WHO guidelines
Cordova et al. (USA) [103] (OS)	General ICU	59	90.2	Alarms, conversations, equipment	Noise levels exceeded WHO limits at all times
Khademi et al. (Iran) [97] (OS)	Various ICUs and emergency wards	60.2	94	Medical alarms, staff and visitor conversations	Average and peak noise levels exceeded international standards
Hsu et al. (Taiwan) [88] (OS)	Postcardiac surgery ICU	59.0	81.3	Medical devices, monitors, alarms	Noise significantly associated with increases in HR, SABP, DABP, MABP; perceived psychological/physiological responses not significantly associated with noise
Dennis et al. (USA) [78] (QeI)	Neuro ICU	NS	83.1	Staff activity, central station noise (phones, pagers, printers), proximity to exits/lounge	Significant reduction in noise and light during day shift QT; increased likelihood of patients being observed asleep (4x more during QT than before). Night shift showed less dramatic but still significant changes
Cardoso Macedo et al. (Brazil) [10] (OS)	General ICU, coronary care unit	64.1	85	Ambient ICU equipment and staff activity during peak hours	Sound levels exceeded recommended levels; no values > 85 dB; no occupational risk but high morbidity concern for patients
Anand et al. (UK) [104] (OS)	General ICU (with conservatory)	59	79.1	Alarms, phones, door bells, general staff activity	Noise levels exceeded WHO guidelines day and night. No significant day/night difference. Bed near nurses’ station was noisiest
Qutub and El‐Said (Saudi Arabia) [105] (OS)	General ICU	60.4	NS	Mechanical alarms, devices, conversations	No significant difference between shifts or weekdays vs weekends
Tsara et al. (Greece) [106] (OM)	General ICU	59.1	NS	Medical equipment alarms, continuous nursing and medical activity.	ICU noise levels consistently above recommended levels; no significant variation across days.
Akansel & Kaymakçi (Turkey) [107] (OS)	Coronary care cnit (Post‐CABG)	65	89	Monitor alarms, conversations, other patients, ER/OR admissions	Prior ICU experience increased sensitivity to noise
Vinodhkumaradithyaa et al. (India) [108] (OS)	General ICU	58.34	62.5	Staff communication, equipment, alarms, vehicular traffic, mobile phones	ICU had the lowest noise level among hospital areas
Christensen (UK) [109] (OS)	General ICU, 9 beds	56.4	80	Staff activity, alarms (inferred)	Statistically significant variation between shifts
Goldenberg et al. (USA) [17] (OS)	Emergency room, ICU, hospital floors; nursing home floors (NH)	64.1	NR	Human activity, technology (monitors, alarms, cleaning devices)	ICU levels higher than EPA limit; staff activity is key source

*Note:* SiS, simulation study.

Abbreviations: ABP, arterial blood pressure; EPA, Environmental Protection Agency; HR, heart rate; NR, not reported; OM, observational (multicenter); OS, observational (single‐center); QeI, quasi‐experimental/interventional; WHO, World Health Organization.

### 3.3. Physiological and Psychological Effects of ICU Noise on Patients

Most healthy adults can tolerate daytime noise levels of 50–55 dBA and nighttime levels of 40–45 dBA without discomfort. However, continuous exposure above 30 dBA and peak noises of 45 dBA may disrupt sleep quality [27–30]. ICU patients are particularly vulnerable due to illness severity, altered sleep–wake cycles, and sensory overload [31]. Excessive and persistent noise activates the hypothalamic–pituitary–adrenal axis and the sympathetic nervous system, elevating cortisol, heart rate, oxygen consumption, and sedation requirements, while also altering melatonin rhythms [1, 7, 32, 33]. These responses may impair recovery. Noise may also increase pain perception and the need for sedatives and analgesics, thereby prolonging mechanical ventilation and ICU stay [7, 33].

Sleep disruption is among the most well‐documented consequences of ICU noise. ICU patients often receive fewer than 5 hours of light, fragmented sleep daily, lacking restorative slow‐wave and REM phases [2, 34–37]. Sounds exceeding 80 dBA have been shown to trigger cortical arousals and awakenings [34, 38–40]. Noise‐induced circadian disruption, including dysregulated melatonin and cortisol secretion, is further exacerbated by artificial lighting and mechanical ventilation, contributing to immune suppression, metabolic dysregulation, and cognitive dysfunction [1, 41–49]. Delirium affects up to 80% of ventilated ICU patients and is strongly associated with environmental noise exposure [7, 50–52]. Alarms and loud conversations in particular have been linked to vivid and distressing patient memories [7, 34].

### 3.4. Impact of ICU Noise on Staff

ICU noise negatively affects staff performance and well‐being. It impairs speech comprehension, memory, and attention, functions critical for safe and effective clinical practice. This contributes to disrupted communication during handovers, reduced intelligibility of verbal instructions and alarms, especially when staff wear personal protective equipment, and delayed response times due to auditory masking [7, 16, 53].

Chronic noise exposure contributes to psychological stress, emotional exhaustion, and irritability. ICU nurses working rotating shifts often report poor sleep, frustration, and reduced job satisfaction, factors directly contributing to burnout [7, 33, 36, 53]. Noise also disrupts tasks requiring sustained focus, such as medication preparation or central line placement. Even moderate background noise has been shown to increase cognitive fatigue and error rates, often without staff’s conscious awareness [20, 54]. A summary of documented effects of ICU noise on healthcare professionals, including cognitive, emotional, and behavioral domains, is presented in Table 2.

**TABLE 2:** Summary of noise‐related effects on ICU staff.

Impact domain	Observed effects	Supporting literature
Cognitive load	Impaired concentration, difficulty hearing and recalling verbal information.	Song and Lee [53] (OM)
		Pal et al. [7] (SR)

Communication	Speech masking, misinterpretation of verbal orders, vocal strain.	Song and Lee [53] (OM)
		Pal et al. [7] (SR)
		Blomkvist et al. [55] (RCT)

Burnout	Irritability, emotional fatigue, job dissatisfaction, stress.	Terzi et al. [56] (OS) Vieira et al. [57] (OM)
		Riemer et al. [58] (QI)
		Wang et al. [59] (OS)
		Ryherd et al. [60] (OS)

Performance	Reduced precision in procedures, increased likelihood of error.	

Workplace culture	Vocal escalation, avoidance of bedside discussion, reduced presence near patients.	Song and Lee [53] (OM)
		Khademi and Imani [61] (SR)

*Note:* OS, observational single‐center; QI, quality improvement study;.

Abbreviations: OM, observational multicenter; RCT, randomized controlled trial/interventional; SR, systematic review.

### 3.5. Effects of Interventions

Several studies have evaluated interventions aimed at reducing ICU noise or mitigating its adverse effects on patients and staff. In one redesign project, the installation of acoustic ceiling tiles and spatial reorganization achieved a 5–7 dBA reduction in ambient sound levels and was associated with improved satisfaction among both patients and staff [62]. Additional architectural strategies, such as the use of double‐glazed windows, padded doors, and the relocation of alarms away from the bedside, have also been reported as effective in reducing noise propagation within the ICU environment [63, 64].

Alarm management protocols have demonstrated significant potential in reducing sound exposure. A review of 11 studies showed that protocol‐driven alarm customization, including individualized threshold settings and delay functions, led to a 15%–35% reduction in alarm volume and improved staff responsiveness [65].

Behavioral interventions have also yielded measurable outcomes. A structured educational campaign directed at ICU staff resulted in an average reduction of 7 dBA in background noise and was associated with improved patient sleep and awareness [66]. Bundled approaches targeting the broader sensory environment have shown additional promise. For example, the “SLEEP” bundle, comprising support, light, environment, eye masks, and earplugs, was associated with improved sleep quality and a 20% reduction in delirium incidence [24]. Similarly, comprehensive frameworks incorporating noise control, circadian rhythm support, and sleep‐promoting strategies have demonstrated additive benefits in diverse ICU settings [7, 67, 68]. Table 3 provides a summary of selected ICU noise‐reduction strategies reported in the literature, categorized by approach and setting, along with their observed impacts.

**TABLE 3:** Summary of noise reduction strategies in the ICU.

Category	Examples	Reported impact	Key references
Architectural	Sound‐absorbing panels, enclosed layouts, acoustic tiles.	5–10 dBA reduction, improved patient sleep and satisfaction	Tronstad et al. [62] (QI)
			Imbriaco et al. [64] (SiS)
			Luetz et al. [69] (RCT)
			Tegnested et al. [25] (OS)
			Blomkvist et al. [55] (RCT)
			Hagerman et al. [70] (RCT)

Technological	Alarm delay settings, visual alerts, smart alarms	Noise reduction, partial persistence postremoval; behavioral effect observed	Vreman et al. [65] (SR)
			Pal et al. [7] (SR)
			Guisasola‐Rabes et al. [71] (QI)
			Plummer et al. [72] (QI)

Behavioral	Quiet hours, staff education, signage	Inconsistent impact, short‐term impact unless reinforced	Souza et al. [66] (QI)
			Jonescu et al. [50] (QI)
			Crawford et al. [13] (RCT)
			Zamani et al. [73] (RCT)
			Tainter et al. [74] (OS)

Multicomponent bundles	SLEEP bundles, integrated environmental and sleep protocols	Improved sleep, reduced delirium, more sustainable outcomes	Kol et al. [24] RCT
			Nannapaneni et al. [75] (QI)
			Patel et al. [76] (RCT)
			Mattingly and Kate [77] (QI)
			Dennis et al. [78] (QI)
			Cmiel et al. [79] (QI)
			Peacock et al. [80] (OS)
			Tainter et al. [74] (OS)
			Xu et al. [81] (RCT)
			Trask et al. [82] (QI)

*Note:* SLEEP, support, light, environment, eye masks, and earplugs. OS, observational (single‐center); QI, quality improvement study; SiS, simulation study.

Abbreviations: RCT, randomized controlled trial/interventional; SR, systematic review.

## 4. Discussion

Noise in ICUs has been most frequently assessed with professional sound level meters certified to IEC 61672 standards, reporting *L*_Aeq_, *L*_max_, and *L*_min_ with A‐weighting [25, 51, 83]. Measurements are usually performed near the patient’s head or in shared areas, continuously or across nursing shifts [6]. Observational methods such as documenting alarms, staff conversations, or equipment use provide complementary information on the frequency and type of disruptive sounds [21, 24, 71]. More recently, smartphone applications have been explored, with acceptable agreement for average noise levels but less reliability for peaks [84]. Other studies have relied on indirect measures, including patient‐reported sleep disturbance, anxiety, or noise annoyance, as well as physiological responses such as heart rate, blood pressure, and cortisol [33, 36, 85], sometimes combined with sleep monitoring [46, 47]. While objective acoustic monitoring remains the reference standard, these complementary approaches highlight the multifaceted impact of ICU noise and underscore the need for standardized protocols to improve methodological consistency and comparability across studies.

Critically ill patients are uniquely vulnerable to the disruptive effects of environmental noise. ICU soundscapes have been linked to fragmented sleep, impaired circadian rhythms, and heightened stress responses [7, 33, 86, 87]. While sleep disruption is common in critically ill patients, noise adds a modifiable burden, especially through unpredictable, high‐pitched alarms or conversations near the patient’s head. Some patients later describe their ICU experience as overwhelming or even traumatic, with noise playing a prominent role in the formation of negative memories [26, 34, 79, 88]. Although interventions such as earplugs or white noise have been tested, evidence for their effectiveness remains mixed [2, 3, 30, 32, 37, 48, 89–91]. Inconsistencies may reflect differences in ICU layout, baseline noise levels, and patient susceptibility. Nonetheless, the clinical significance of environmental noise remains underappreciated in practice and poorly integrated into patient‐centered care models.

Excessive noise also compromises staff well‐being and performance. Staff may unconsciously raise their voices to compete with ambient noise, creating a feedback loop that amplifies acoustic strain [7, 20, 54]. Long‐term exposure contributes to emotional exhaustion, irritability, and job dissatisfaction. These factors, in turn, fuel burnout [36, 56, 92]. Under persistent strain, clinicians may adopt task‐oriented care styles, shortening patient interactions or avoiding complex conversations. While such adaptations can preserve efficiency, they risk eroding empathy and diminishing the human dimension of ICU practice [16, 53].

The causes of ICU noise are deeply embedded in the physical and social fabric of care delivery. Behavioral norms, such as unfiltered conversations, shift handovers at the bedside, and informal discussions, generate frequent acoustic peaks. Proximity between staff work areas and patient beds further amplifies noise propagation [29, 30, 34]. Importantly, these patterns often go unchallenged, even when awareness campaigns are introduced [53, 62].

**FIGURE 2:**
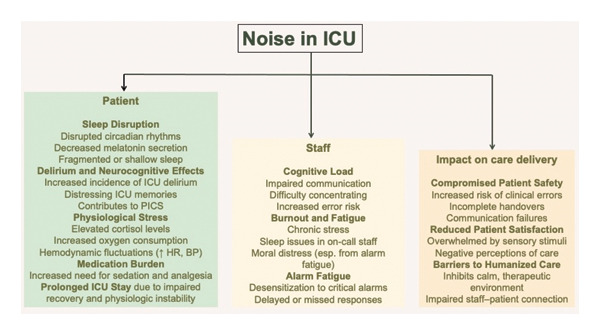
Conceptual model of the impact of ICU noise on patients, staff, and care delivery. Noise in the intensive care unit arises from multiple sources and affects three main domains, patients, staff, and the delivery of care. ICU, intensive care unit; PICS, postintensive care syndrome; HR, heart rate; BP, blood pressure.

Alarms remain a dominant source of acoustic burden. Despite efforts to standardize and filter alerts, thresholds are rarely personalized, and alarm fatigue is widespread. Staff frequently encounter high alarm densities with limited capacity to distinguish clinically significant signals. False positives are common, and the prioritization of alarm fidelity over patient comfort often reflects a deep‐rooted culture of risk aversion rather than evidence‐based practice [1, 7, 16, 93–95]. The multidimensional impact of ICU noise on patients, staff, and care systems is summarized in Figure 2, which highlights the interplay between physiological stress, cognitive burden, and care quality.

Physical design plays a pivotal role in shaping the ICU soundscape. Open‐plan units with hard surfaces allow sound to travel and reverberate freely, while design interventions such as acoustic ceiling tiles, padded doors, and sound‐absorbing materials can help reduce peak levels and reverberation times [15, 62–64]. Yet, such architectural solutions are often feasible only during construction or major renovation, limiting their real‐world applicability. Acoustic modeling studies have shown that even modest improvements in spatial layout or surface materials can yield perceptible reductions in noise exposure. Noise can also originate from outside the ICU, such as nearby staff lounges, cafeterias, or hallway traffic. These peripheral sources often go unaddressed in noise management strategies but can substantially raise the overall acoustic burden [7, 84]. Incorporating these insights into ICU design standards could offer long‐term gains in patient comfort and staff resilience.

A wide array of interventions has been tested to reduce ICU noise, ranging from behavioral protocols to bundled environmental strategies. While some show promise, most are limited by short follow‐up periods, a lack of standardization, and inconsistent outcomes across settings. Behavioral interventions, such as quiet‐time protocols, noise‐awareness signage, and decibel monitors, can improve staff awareness, but their effects often diminish without reinforcement [50, 66, 74]. Alarm management strategies, including personalized thresholds or delay functions, are effective in theory but require cultural and infrastructural alignment. More advanced tools, such as context‐aware alarms or wearable noise monitors, remain in early‐stage adoption and are often resource‐intensive [65]. Multicomponent bundles that address multiple environmental stressors, such as light and noise, appear more promising. These holistic approaches align better with ICU realities and are more likely to produce sustainable outcomes, although further study is needed to confirm their effectiveness in diverse settings [7, 24, 67, 68].

Although a wide range of noise‐reduction strategies has been evaluated, the available evidence does not support a formal ranking of interventions by effectiveness. Individual strategies differ substantially in design, targets, outcomes, and implementation context, precluding direct comparison. Nevertheless, qualitative synthesis across studies suggests that single, isolated interventions such as signage, quiet hours, or standalone educational campaigns tend to produce modest and often transient effects, particularly when reinforcement is lacking. While staff education increases awareness of noise sources and their clinical consequences, educational efforts alone rarely translate into durable behavioral change unless they are embedded within structured quality‐improvement frameworks, supported by leadership, and reinforced through ongoing feedback. In contrast, multicomponent and system‐level approaches, integrating alarm management, sustained staff engagement, environmental modifications, educational reinforcement, and sleep‐promoting practices, appear more consistently associated with sustained improvements in both acoustic conditions and clinically relevant outcomes. Architectural interventions demonstrate robust noise attenuation but are often limited by feasibility and cost, whereas behavioral and technological measures require continuous cultural and organizational support to maintain effectiveness. These findings highlight that the relative impact of noise‐reduction strategies is context‐dependent and that durable improvements are most likely achieved through integrated, multimodal frameworks rather than any single dominant intervention [65, 96].

Despite decades of evidence documenting the adverse effects of excessive noise on patients and staff, ICU sound levels remain persistently elevated. This apparent paradox reflects not a lack of awareness but a convergence of cultural norms, architectural constraints, alarm design priorities, and limited sustainability of behavior‐based interventions. Beyond individual interventions, cultural inertia remains a barrier. In some ICUs, noise is interpreted as a sign of vigilance or activity, and silence may be perceived as neglect. Noise mitigation is rarely framed as a quality or safety priority. Without formal accountability, benchmarks, designated responsibilities, or routine monitoring, meaningful change is difficult to sustain [7, 50]. Heterogeneity in research methods further complicates implementation. Studies often differ in noise metrics, sampling durations, and reporting standards, making comparisons difficult and guidelines inconsistent [34, 84, 97]. For noise mitigation to become a clinical standard, both scientific consensus and policy support will be necessary.

Several pathways could strengthen the evidence base and operational integration of ICU noise control. First, large multicenter trials are needed to evaluate bundled interventions with long‐term follow‐up and standardized outcome measures. These studies should examine not only sound levels but also clinical endpoints such as delirium, sedation requirements, and staff burnout. Second, acoustic engineering principles must be embedded in ICU design from the outset, with attention to noise zoning, surface treatments, and spatial separation between patients and staff work areas [62]. Third, innovation should be encouraged around smart alarm systems, real‐time feedback tools, and adaptive soundscapes responsive to circadian rhythms or patient status [65]. Fourth, behavioral change should be driven by local leadership and cultural modeling. Involving nurse champions, patients, and families in quiet‐time policies can enhance shared responsibility and normalize new practices [79]. Finally, policy frameworks should explicitly include noise control as part of ICU quality improvement. Integrating acoustic benchmarks into accreditation or performance metrics could provide the institutional leverage needed for sustained improvement [13].

To enhance the practical relevance of this narrative review, we summarize below a set of pragmatic considerations that may support noise reduction initiatives in the ICU. These points are not intended as prescriptive recommendations but rather as implementation‐oriented insights derived from the available literature, acknowledging the heterogeneity of interventions and the context‐dependent nature of their effectiveness.

### 4.1. Potential Interventions

• Prioritize multimodal approaches combining staff education, alarm management, and environmental measures rather than isolated interventions.
• Review and optimize alarm settings, including volume, thresholds, and escalation protocols, to reduce nonactionable alarms and alarm fatigue.
• Promote staff awareness of noise‐generating behaviors through targeted education and feedback, recognizing that sustained effects require institutional reinforcement.
• Implement environmental strategies where feasible, such as sound‐absorbing materials, equipment maintenance, and spatial zoning.
• Consider patient‐centered measures, including earplugs or music interventions, as adjuncts rather than standalone solutions.

### 4.2. Measuring Impact

• Use standardized acoustic metrics (e.g., *L*_Aeq_ and *L*_max_) and clearly define measurement locations and time periods.
• Combine acoustic data with clinically relevant outcomes, such as sleep quality, delirium incidence, or staff‐reported outcomes.
• Where feasible, follow‐up measurements over time can help assess whether observed improvements are sustained beyond short‐term effects.
• Embed noise monitoring within broader quality improvement frameworks rather than as one‐off audits.

### 4.3. Limitations

This narrative review was not designed to achieve exhaustive literature coverage or formal minimization of selection bias but rather to provide an integrative and interpretive synthesis of heterogeneous evidence related to ICU noise, its clinical impact, and mitigation strategies. This methodological choice allows for contextual discussion across disciplines, care settings, and intervention types; however, it inherently carries limitations, including potential selection bias and the absence of standardized quality appraisal or quantitative synthesis.

There is considerable heterogeneity in how ICU noise is measured across studies. Differences in instrumentation, data collection protocols, and reporting metrics (e.g., *L*_Aeq_, *L*_max_, and peak frequency) complicate comparisons and preclude meta‐analytic synthesis. Additionally, many studies report only short sampling durations or lack continuous monitoring, which may underestimate the true variability and burden of noise exposure. These methodological differences complicate direct comparisons across studies and further support the narrative and interpretive approach adopted in this review.

Another limitation is the relative paucity of high‐quality interventional studies. While various strategies for noise reduction have been proposed and piloted, few have undergone rigorous, multicenter evaluation with long‐term follow‐up. This limits the ability to draw firm comparative conclusions regarding the effectiveness and sustainability of individual interventions.

Consistent with the narrative scope of this review, most available evidence focuses on adult ICU populations, with limited data on specific subpopulations such as the elderly, patients with cognitive impairment, or those in palliative care, who may be differentially affected by environmental noise. Similarly, staff‐related outcomes are often underreported or qualitatively assessed, reducing insight into the full occupational impact of noise.

## 5. Conclusion

Noise in the ICU is a pervasive and persistent challenge with far‐reaching consequences. It disrupts patient sleep, contributes to delirium, elevates physiological stress responses, and impairs communication and performance among staff. Despite decades of research and a growing body of evidence linking noise to adverse outcomes, ICU sound levels remain consistently above recommended thresholds. While many strategies for noise control show promise, their effectiveness is often limited by narrow implementation, a lack of sustainability, and failure to address noise as a systemic issue. The way forward demands a paradigm shift: one that treats the ICU acoustic environment not as a peripheral comfort issue but as a core dimension of patient safety and staff well‐being. Future initiatives must embrace interdisciplinary collaboration, evidence‐based design, and continuous quality improvement frameworks. With targeted research, smarter technologies, and committed cultural change, it is possible to create quieter, safer, and more humane ICUs.

## Author Contributions

Charikleia S. Vrettou contributed to the investigation, visualization, writing the original draft, and editing. Panagiotis T. Koliotsis contributed to writing the original draft, editing, and visualization. Spyretta Golemati contributed to conceptualization, methodology, and editing. Giorgos Mastorakis contributed to the investigation, resources, and editing. Vassiliki Karaviti contributed to the investigation, resources, and visualization. Sofia Mavromati contributed to the investigation, resources, and editing. Maria Tagara contributed to resources, methodology, and editing. Maria P. Papadopoulou contributed to conceptualization, editing, methodology, and supervision. Ioanna Dimopoulou contributed to conceptualization, supervision, and editing.

## Funding

Parts of the research work described in this paper were conducted within the project “ER‐NOISE MAPPING—Mapping and investigating the effects of patient exposure to environmental noise in Intensive Care Units (ICUs)” funded by the EMPIRIKION FOUNDATION under the grant “Financial aid in memory of Miltiades Empirikos 2024.”

## Disclosure

The authors have nothing to report.

## Ethics Statement

Since this review only includes data from previously published sources, there was no need for ethical approval. The ethical guidelines stated in Elsevier’s Publishing Ethics Policy were followed.

## Conflicts of Interest

The authors declare no conflicts of interest.
